# Supplementary material for: Mechanism of genome instability mediated by human DNA polymerase mu misincorporation
Source: Nat Commun. 2021 Jun 18;12:3759. doi: 10.1038/s41467-021-24096-7 (PMC8213813; doi:10.1038/s41467-021-24096-7)

Mechanism of genome instability mediated by human DNA polymerase mu misincorporation

Miao Guo[1,2], Yina Wang[1,2], Yuyue Tang[1,2], Zijing Chen[1,2], Jinfeng Hou[1,2], Jingli Dai[1,2], Yudong Wang[1,2], Liangyan Wang[1,2], Hong Xu[1,2], Bing Tian[1,2], Yuejin Hua[1,2 *] & Ye Zhao[1,2 *]

[1.] Institute of Biophysics, College of Life Sciences, Zhejiang University, Hangzhou, Zhejiang, China

[2.] MOE Key Laboratory of Biosystems Homeostasis & Protection, Zhejiang University, Hangzhou, Zhejiang, China

* To whom correspondence should be addressed. Tel: 86-571-86971279; fax: 86-571-86971703; email: Z. Y. (yezhao@zju.edu.cn)

Correspondence may also be addressed to yjhua@zju.edu.cn

Keywords: polymerase mu; misincorporation; manganese; gap filling; mutagenesis

Summary of supplemental information

Supplementary Table 1. Data collection and refinement statistics

Supplementary Table 2. Kinetic parameters of dGTP misincorporation opposite 1-nt gapped DNA containing templating T by mutant pol μ

Supplementary Table 3. DNA used for crystallization and kinetic measurements

Supplementary Figure 1. Extension after the T:G mispair

Supplementary Figure 2. Overall structure of Pol μ binary complex and ternary complexes

Supplementary Figure 3. dGMPNPP and the 3′-end dA in the Complex 3

Supplementary Figure 4. Superposition of Complex 2 flipped conformation and correct insertion structures

Supplementary Figure 5. Templating T in the Complex 4

Supplementary Figure 6. Superposition of Complex 4 (Q441A) and Complex 5

Supplementary Table 1: Data collection and refinement statistics

	Binary complex	Complex 1	Complex 2	Complex 3
Protein / Metal	WT / -	WT / Mg	WT / Mg	WT / Mn
DNA	1-nt gap	2-nt gap	1-nt gap	1-nt gap
PDB Code	7CO6	7CO8	7CO9	7COA
Data collection				
Space group	$P2_12_12_1$	$P2_12_12_1$	$P2_12_12_1$	$P2_12_12_1$
Cell dimensions				
a, *b*, *c* (Å)	60.18	60.31	60.08	59.95
	62.59	69.20	68.54	68.57
	117.79	117.86	110.82	110.81
Wavelength (Å)	0.9792	0.9792	0.9792	0.9792
Resolution (Å)	30-1.9	30-1.7	30-1.6	30-1.7
R_{sym} (%)	4.9 (53.4)	7.7 (52.9)	3.9 (26.4)	5.9 (37.9)
$I/\sigma I$	17.9 (3.5)	13.4 (2.9)	22.1 (3.9)	17.3 (3.8)
Completeness (%)	99.4 (99.8)	99.2 (94.8)	98.7 (93.0)	99.5 (96.8)
Redundancy	5.4 (5.6)	5.3 (4.3)	5.1 (3.3)	5.4 (4.5)
Refinement				
Resolution (Å)	30-1.9	30-1.7	30-1.6	30-1.7
No. reflections	35807	55307	61235	51199
R_{work}/R_{free}	17.9/21.4	17.9/19.5	17.1/19.6	17.5/19.9
No. atoms				
Protein/DNA	2657/343	2777/363	2761/384	2734/384
Ligand/ion	6/2	31/4	62/3	62/3
Water	190	383	379	361
B-factors				
Protein/DNA	39.4/30.5	24.0/27.4	25.3/24.7	25.5/25.6
Ligand/ion	44.2/37.3	26.1/24.3	34.2/20.5	28.8/17.0
Water	43.3	35.0	37.5	36.7
R.m.s deviations				
Bond lengths (Å)	0.005	0.010	0.012	0.012
Bond angles (°)	0.718	0.957	1.077	1.077
Ramachandran statistics				
Favored (%)	97.2	96.8	97.8	97.5
Allowed (%)	2.8	3.2	2.2	2.5
Outliers (%)	0	0	0	0

	Complex 4	Complex 5	Post-insertion complex
Protein / Metal	Q441A / Mg	Q441A/K438A / Mg	Q441A/K438A / Mg
DNA	1-nt gap	1-nt gap	1-nt gap
PDB Code	7COB	7COC	7COD
Data collection			
Space group	$P2_12_12_1$	$P2_12_12_1$	$P2_12_12_1$
Cell dimensions			
a, *b*, *c* (Å)	59.76	59.96	60.18
	68.34	68.56	68.52
	110.60	110.54	111.91
Wavelength (Å)	0.9792	0.9792	0.9792
Resolution (Å)	30-1.8	30-1.9	30-1.8
R_{sym} (%)	4.9 (57.6)	5.7 (60.4)	3.6 (30.3)
$I/\sigma I$	16.9 (2.6)	16.3 (3.0)	24.2 (2.5)
Completeness (%)	99.7 (97.1)	98.8 (97.1)	95.1 (71.5)
Redundancy	5.4 (5.4)	5.4 (5.3)	4.7 (2.2)
Refinement			
Resolution (Å)	30-1.8	30-1.9	30-1.8
No. reflections	42944	36699	43625
R_{work}/R_{free}	18.0/20.4	18.2/21.1	18.6/21.1
No. atoms			
Protein/DNA	2690/402	2695/383	2716/426
Ligand/ion	31/3	31/3	37/4
Water	237	256	348
B-factors			
Protein/DNA	34.4/35.1	33.0/34.5	28.3/32.3
Ligand/ion	36.8/34.1	34.9/30.3	39.4/24.7
Water	41.4	39.5	38.2
R.m.s deviations			
Bond lengths (Å)	0.010	0.010	0.010
Bond angles (°)	1.008	1.030	0.983
Ramachandran statistics			
Favored (%)	96.7	97.6	97.9
Allowed (%)	3.3	2.4	2.1
Outliers (%)	0	0	0

Values in parentheses refer to the highest resolution shell.

R factor = $\Sigma||F(obs)- F(calc)||/\Sigma|F(obs)|$.

Rfree = R factor calculated using 5.0% of the reflection data randomly chosen and omitted from the start of refinement.

Supplementary Table 2: Kinetic parameters of dGTP misincorporation opposite 1-nt gapped DNA containing templating T by mutant pol μ

Enzyme	dNTP	k_m (μM)	k_{cat} (min^{-1})	k_{cat}/k_m ($min^{-1}\cdot\mu M^{-1}$)	f_{mis} *
WT	dATP	3.1±0.3	$236.2\times10^{-3}\pm5.3\times10^{-3}$	76.6×10^{-3}	6.4×10^{-2}
	dGTP	5.7±0.5	$28.3\times10^{-3}\pm0.6\times10^{-3}$	4.9×10^{-3}	
Q441E	dATP	8.6±0.6	$398.5\times10^{-3}\pm\times8.6\times10^{-3}$	46.3×10^{-3}	2.2×10^{-2}
	dGTP	79.2±9.5	$79.8\times10^{-3}\pm\times2.8\times10^{-3}$	1.0×10^{-3}	
Q441N	dATP	3.6±0.5	$188.6\times10^{-3}\pm\times7.1\times10^{-3}$	52.4×10^{-3}	5.2×10^{-2}
	dGTP	4.5±0.7	$12.0\times10^{-3}\pm\times0.4\times10^{-3}$	2.7×10^{-3}	
K438R	dATP	2.8±0.3	$354.0\times10^{-3}\pm7.6\times10^{-3}$	126.4×10^{-3}	2.5×10^{-2}
	dGTP	12.0±1.8	$37.9\times10^{-3}\pm1.0\times10^{-3}$	3.2×10^{-3}	
K438Q	dATP	2.0±0.2	$93.2\times10^{-3}\pm1.6\times10^{-3}$	46.6×10^{-3}	1.9×10^{-2}
	dGTP	1.9±0.1	$1.7\times10^{-3}\pm0.02\times10^{-3}$	0.9×10^{-3}	

* f_{mis} is the relative efficiency of misincorporation expressed as the ration of $[k_{cat}/K_{m\mathrm{dGTP}}]/[k_{cat}/K_{m\mathrm{dATP}}]$

Supplementary Table 3: DNA used for crystallization and kinetic measurements

Primers	Sequence (5′→3′)
Crystallization	
1-nt gapped DNA	template strand: CGGCTTACG<br>upstream primer: CGTA<br>downstream primer: pGCCG (5′-phosphorylated)
2-nt gapped DNA	template strand: CGGCTTTACG<br>upstream primer: CGTA<br>downstream primer: pGCCG (5′-phosphorylated)
Primer extension	
1-nt gapped DNA	template strand: CTCCGTCGTCCGGCTTACGTCAGTCTGAC<br>upstream primer: FAM-GTCAGACTGACGTA (6-FAM-5′-labeled)<br>downstream primer: pGCCGGACGACGGAG (5′-phosphorylated)
2-nt gapped DNA	template strand: CTCCGTCGTCCGGCTTTACGTCAGTCTGAC<br>upstream primer: FAM-GTCAGACTGACGTA (6-FAM-5′-labeled)<br>downstream primer: pGCCGGACGACGGAG (5′-phosphorylated)

Supplemental Figure Legends

Supplementary Figure 1: Extension after the T:G mispair. Reactions were carried out at a substrate:enzyme molar ratio of 25:1, with a single type or a mixture of all four dNTPs (total concentration of 0.5 mM) at 37°C for 7 min as described in the Method section. Bands beyond the product of dGTP misincorporation are indicated by red arrowheads. The reactions were independently repeated three times with similar results.

WT + Mg^{2+}

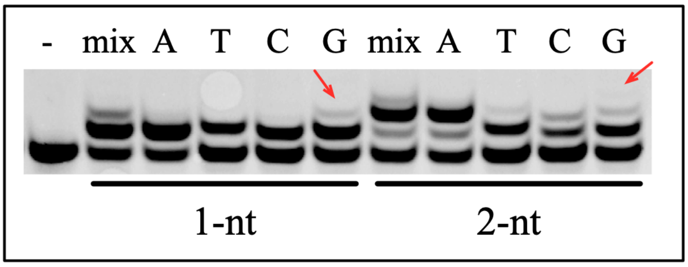

Supplementary Figure 2: Overall structure of Pol μ binary complex and ternary complexes. (a) Superposition of seven pol μ structures solved in the current study. Protein domains of Pol μ, dGMPNPP, and Mg^{2+} ions are labeled and shown in distinct colors. Loop1 region (grey) with different trajectory in Complex 1 and Complex 2 is labeled and indicated by black arrowheads. (b) Superposition of DNA and incoming nucleotides in Complexes 2-5, post-insertion complex, and the correct insertion complex (PDB: 4M04) of Pol μ.

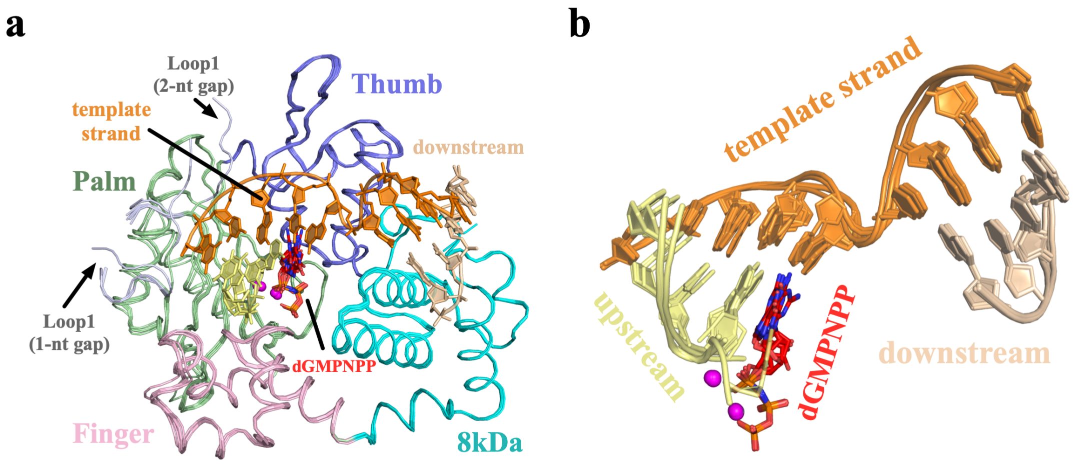

Supplementary Figure 3: dGMPNPP and the 3′-end dA in the Complex 3. Reaction-ready conformation (Conformation A, yellow) and the flipped conformation (Conformation B, light blue) of the dGMPNPP and 3′-end dA are superimposed with the 2Fo-Fc (silver; contoured at 0.7σ) and Fo-Fc omit map (green; contoured at 2.5σ), which were calculated without the flipped conformation.

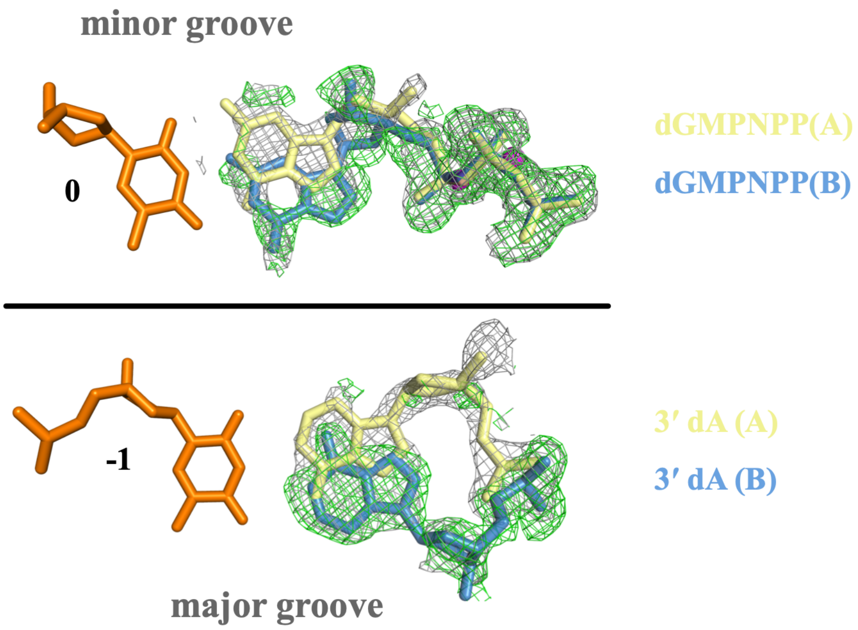

Supplementary Figure 4. Superposition of Conformation B in Complex 2 (orange and cyan) and correct insertion (PDB: 4M04, white) structures of Pol μ. Hydrogen bonds are indicated by dashed lines. The black arrowheads indicate the relative movements of these two conformations.

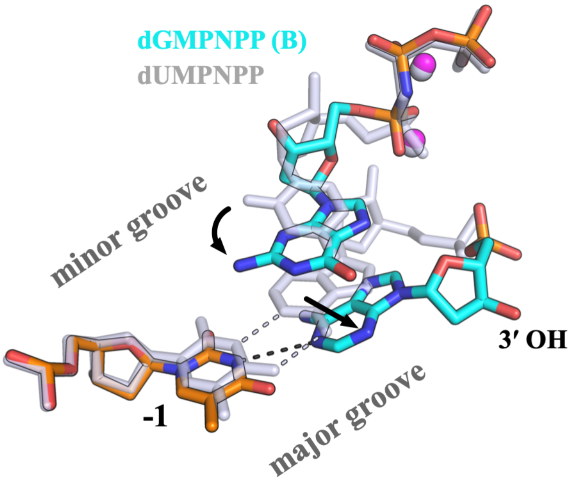

Supplementary Figure 5: Templating T in the Complex 4. Canonical conformation (Conformation A, orange) and the shifted conformation (Conformation B, white) of the Templating T are superimposed with the 2Fo-Fc (silver; contoured at 0.8σ) and Fo-Fc omit map (green; contoured at 3.0σ), which were calculated without the shifted conformation (Conformation B). Red arrowhead indicates the movement between the phosphate backbone of two DNA conformations.

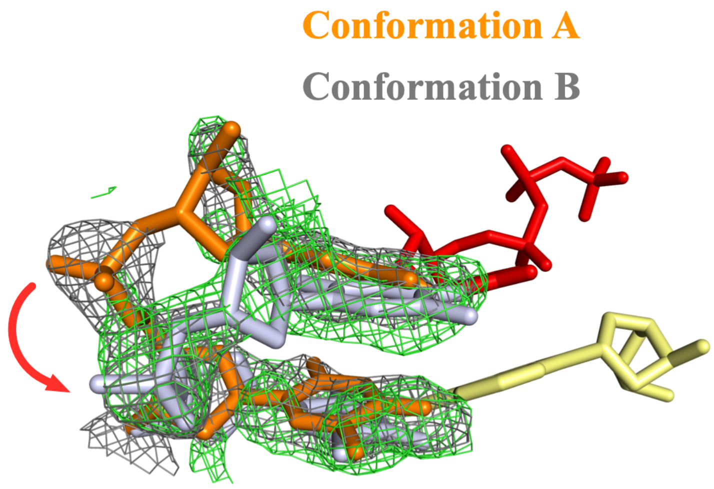

Supplementary Figure 6: Superposition of Complex 4 (Q441A) and Complex 5. Complex 4 structure is colored white with shifted conformation of templating T (T0) in orange. Complex 5 is shown in distinct colors with further shifted conformation of templating T (T0) in cyan. Templating T in canonical conformation in Complexes 4 and 5 are colored white.

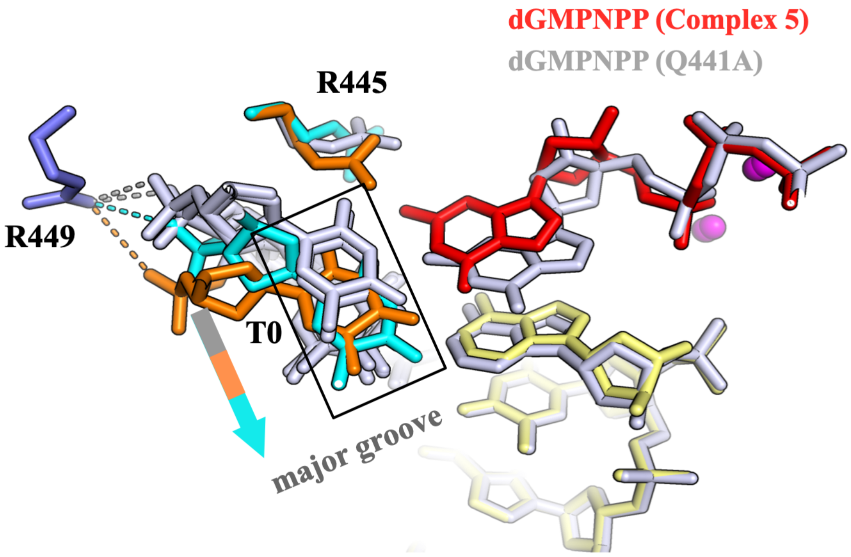

Supplement: Supplementary file 1 — Supplementary Information [file 41467_2021_24096_MOESM1_ESM.pdf]
